# Geochemistry and Microbiology Predict Environmental Niches With Conditions Favoring Potential Microbial Activity in the Bakken Shale

**DOI:** 10.3389/fmicb.2020.01781

**Published:** 2020-07-30

**Authors:** Kara Tinker, James Gardiner, Daniel Lipus, Preom Sarkar, Mengling Stuckman, Djuna Gulliver

**Affiliations:** ^1^National Energy Technology Laboratory, Pittsburgh, PA, United States; ^2^Oak Ridge Institute for Science and Education, Oak Ridge, TN, United States; ^3^Leidos Research Support Team, National Energy Technology Laboratory, Pittsburgh, PA, United States; ^4^Section of Geomicrobiology, GFZ German Research Centre for Geosciences, Potsdam, Germany

**Keywords:** Bakken Shale, hydraulic fracturing, hydrocarbon, qPCR, 16S rRNA gene amplicon sequencing, microbiome, produced water, unconventional

## Abstract

The Bakken Shale and underlying Three Forks Formation is an important oil and gas reservoir in the United States. The hydrocarbon resources in this region are accessible using unconventional oil and gas extraction methods, including horizontal drilling and hydraulic fracturing. However, the geochemistry and microbiology of this region are not well understood, although they are known to have major implications for productivity and water management. In this study, we analyzed the produced water from 14 unconventional wells in the Bakken Shale using geochemical measurements, quantitative PCR (qPCR), and 16S rRNA gene sequencing with the overall goal of understanding the complex dynamics present in hydraulically fractured wells. Bakken Shale produced waters from this study exhibit high measurements of total dissolved solids (TDS). These conditions inhibit microbial growth, such that all samples had low microbial loads except for one sample (well 11), which had lower TDS concentrations and higher 16S rRNA gene copies. Our produced water samples had elevated chloride concentrations typical of other Bakken waters. However, they also contained a sulfate concentration trend that suggested higher occurrence of sulfate reduction, especially in wells 11 and 18. The unique geochemistry and microbial loads recorded for wells 11 and 18 suggest that the heterogeneous nature of the producing formation can provide environmental niches with conditions conducive for microbial growth. This was supported by strong correlations between the produced water microbial community and the associated geochemical parameters including sodium, chloride, and sulfate concentrations. The produced water microbial community was dominated by 19 bacterial families, all of which have previously been associated with hydrocarbon-reservoirs. These families include *Halanaerobiaceae*, *Pseudomonadaceae*, and *Desulfohalobiaceae* which are often associated with thiosulfate reduction, biofilm production, and sulfate reduction, respectively. Notably, well 11 was dominated by sulfate reducers. Our findings expand the current understanding of microbial life in the Bakken region and provide new insights into how the unique produced water conditions shape microbial communities. Finally, our analysis suggests that produced water chemistry is tightly linked with microbiota in the Bakken Shale and shows that additional research efforts that incorporate coupled microbial and geochemical datasets are necessary to understand this ecosystem.

## Introduction

Hydrocarbon resources represent an important global energy source, currently providing over 75% of primary energy in the United States (U.S. Department of Energy et al., 2020b), and also play an essential role in other countries including Canada and the United Kingdom (Stevens, 2013; Elliott et al., 2014; Rivard et al., 2014). The majority of fossil fuels are currently extracted from unconventional wells using advanced technologies, including horizontal drilling and hydraulic fracturing (U.S. Department of Energy et al., 2020a). Horizontal drilling provides access to low-permeability target formations that were previously inaccessible using conventional, vertical drilling methods. This has resulted in rapidly increased amounts of produced oil and gas from new and established plays across the last decade (U.S. Department of Energy et al., 2020b). Hydraulic fracturing occurs when large volumes of water (5,000–20,000 m^3^) are injected into the subsurface through the horizontally drilled well, increasing the subsurface pressure and fracturing the target formation to release hydrocarbons from the shale (Arthur et al., 2008; Gregory et al., 2011).

Tight oil reservoirs such as the Bakken Formation and underlying Three Forks Formation in North Dakota are one of the impermeable shale plays now accessible through horizontal drilling and hydraulic fracturing. However, these unconventional extraction techniques have introduced new water management and infrastructure challenges raising various operational, economic, and environmental issues for unconventional drilling (Gregory et al., 2011; Orangi et al., 2011). Hydraulically fractured wells generate billions of gallons of produced water each year (Horner et al., 2016) which contain high concentrations of salt (Gregory et al., 2011; Barbot et al., 2013; Murali Mohan et al., 2013; Cluff et al., 2014; Lipus et al., 2018; Wang et al., 2019), metals (Gregory et al., 2011; Barbot et al., 2013), and organics (Strong et al., 2014; Akyon et al., 2019) making management, handling, and disposal of these produced waters difficult and expensive. Another major challenge is the presence of various kinds of microorganisms, which may contribute to the corrosion of well-components, including casing and pipes, and reservoir souring (Struchtemeyer and Elshahed, 2012; Mohan et al., 2014; Stringfellow et al., 2014; Daly et al., 2016; Torres et al., 2016; Lipus et al., 2017, 2018). Consequently, microbial activities in hydraulic fracturing may result in operational interruptions, negatively impact hydrocarbon recovery, and create safety and environmental concerns.

Understanding the geochemical and microbial characteristics of produced water from hydraulically fractured wells is essential for effective produced water management and microbial growth control. The Bakken Shale is currently the second most productive oil region in the United States (U.S. Department of Energy et al., 2020c) and contains reserves of 7.4 billions barrels of oil and 190 billion m^3^ of natural gas (Gaswirth, 2013; Plummer et al., 2013), therefore it presents an important study and research target in this field. Previous geochemistry-focused studies report that Bakken Shale produced water is characterized by high total dissolved solids (TDS) (150,000–350,000 mg/L) compared to other active regions (Stepan et al., 2010; Kurz et al., 2016; Thyne and Brady, 2016; Lipus et al., 2018) which have TDS concentrations as low as 680 mg/L (Barbot et al., 2013). Overall, less than 30 studies have examined the microbial community in hydraulically fractured wells, contributing to the identification of novel and shale-specific organisms. These studies demonstrate that produced water is dominated by halophilic, anaerobic, and aerobic groups of microbes, revealing unique metabolic survival strategies (Daly et al., 2016; Liang et al., 2016; Lipus et al., 2017). Only four of these studies have contained samples from the Bakken Shale, with a total of 25 wells represented from this region (Strong et al., 2014; An et al., 2017; Lipus et al., 2018; Wang et al., 2019). The results from these studies are variable. However, previous work suggests shale-associated waters have unique geochemical- and microbial- profiles impacted by factors including season, geographic region, biocidal treatments, well age, and the shale’s post depositional history (Davis et al., 2012; Struchtemeyer et al., 2012; Vikram et al., 2014; Akob et al., 2015; Hull et al., 2018; Lipus et al., 2018; Wang et al., 2019). Therefore, the discrepancy across the three Bakken Shale-focused studies is both unsurprising and highlights the need for additional work that incorporates coupled microbial and geochemical datasets within this region.

Our overall goal is to expand on existing knowledge of the geochemistry and microbiology in hydraulically fractured wells, which will increase insight into the complex geochemical and microbiological dynamics present in these systems. In this study, we analyzed the produced water from 14 unconventional wells in the Bakken and Three Forks Formation. We measured TDS, dissolved organic carbon (DOC), pH, and geochemical data for each produced water sample, and compared the geochemical trends with Bakken data available from the U.S. Geological Survey Produced Water Database (USGS PWDB) (Blondes et al., 2019). Quantitative PCR (qPCR) and 16S rRNA gene amplicon sequencing were used to estimate microbial loads and characterize the microbial community of produced water samples, when possible. Finally, we measured correlations between geochemical and microbial data in order to identify which factors shape the Bakken-associated microbiome. These results provide additional knowledge about the Bakken region, provide valuable context for previous Bakken Shale microbiome studies, and will ultimately be used to improve waste water management and microbial growth control practices in the oil and gas industry.

## Materials and Methods

### Sampling

**FIGURE 1:**
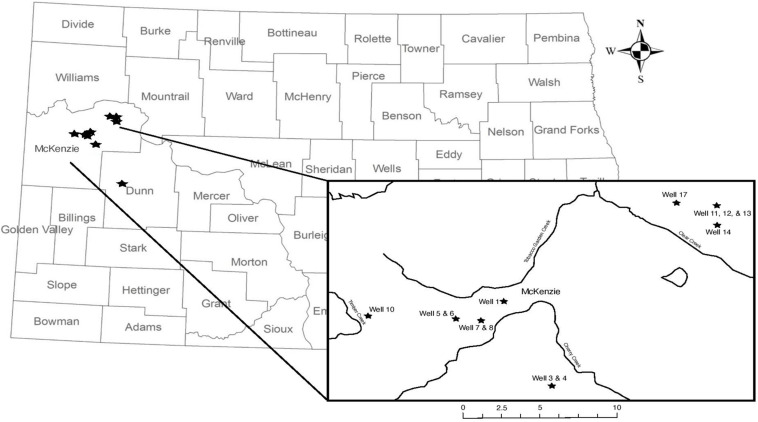
Map of wellhead sampling locations; wellhead location does not indicate the relative size or location of the oil reservoir. Well 18 is located in Dunn County and is not featured on the inset map. Information about well characteristic, including well depth, are found in Table 1.

Produced water samples were collected from 14 hydraulically fractured wells in North Dakota actively producing oil from the Bakken Formation or the underlying Three Forks Formations at the time of sampling in May 2018 (Figure 1). As production data from these formations is typically reported together, we refer to the sampled region as the Bakken Shale or Bakken region throughout this manuscript. Each produced water sample was obtained from the associated three-phase separator, which separates hydrocarbons from the remaining fluids. Separators are often the closest available sampling point to the wellhead and have been used in previous studies (Cluff et al., 2014; Mohan et al., 2014; Lipus et al., 2017). Samples were collected in unused plastic carboys that were pre-rinsed with sample waters and allowed between 1 and 3 h to settle into distinguishable oil and water phases in the sealed container. A portion of the water phase was also collected in a sterile 1 L nalgene bottle and immediately placed on dry ice until arrival in the lab for microbiology analysis. Dry ice was replenished as needed, such that samples remained frozen until they arrived in the lab. Upon arrival in the lab, samples were stored according to previously described guidelines at −20°C until microbial processing (Lipus et al., 2017, 2018, 2019). The remaining water phases were then passed through glass wool cartridges under gravity and then a 0.45 μm inline filter (EnviroTech, Salt Lake City, UT, United States), minimizing oxygen exposure in a closed loop. This filtered water was collected and measured for pH using a Horiba multimeter (Horiba, Edison, NJ, United States) and titrated for alkalinity using a Hach digital titrator (Hach, Loveland, CO, United States). Samples were collected with zero-headspace in Shimadzu TOC vials and transported on ice for total organic carbon (TOC) analysis. A 30 mL volume of the filtered water was acidified to 2% by volume using nitric acid and transported on ice for inductively coupled plasma optical emission spectroscopy (ICP-OES). Finally, a 15 mL volume of the filtered water was passed through a second 0.22 μm sterile polyethersulfone filter (Millipore, Inc.) and shipped on ice for ion chromatography (IC) measurement.

### Chemical Analysis

Major cations and anions were detected using ion chromatography (IC) on a ThermoFisher (Thermofisher, Waltham, MA, United States) ICS-5000+ with AS11-HC column for anion quantification and CS16 column for cation quantification. All samples were run in triplicate and the standard error of IC measurements reported here was less than 3%. Additionally, every 10–20 samples, a cation/anion control sample (Sigma Aldrich, St Louis, MO, United States) with known certified concentrations was added during measurements and all control samples demonstrated an accuracy within 95–105%.

Trace metals were analyzed using inductively coupled plasma optical emission spectroscopy (ICP-OES) on an Optima 7300 DV (Perkin Elmer, Waltham, MA, United States), which is a dual-view spectrometer with solid state SCD detectors. U.S. EPA Method 6010D was employed for analysis with one duplicate, one standard recovery, and one spike recovery. Sample solutions were nebulized using a glass Seaspray concentric nebulizer and a glass Baffled Cyclonic spray chamber (Glass Expansion, Pocasset, MA, United States) using 5 ppm yttrium as an online internal standard. Calibration standards were purchased from Inorganic Ventures (Christiansburg, VA, United States) and are traceable to NIST standard reference materials.

Total organic carbon (TOC) was analyzed with a Shimadzu TOC Analyzer (Model: TOC-LCSN) for non-purgeable organic carbon (NPOC) and total inorganic carbon (TIC). Average values from 3 to 5 replicates were reported with <2% precision (%RSD), and quality control samples for every 10–12 samples demonstrated consistent accuracy within 95–105%.

### DNA Extraction

We extracted DNA from the samples using a modified version of the DNeasy Powersoil kit (Qiagen, Hilden, Germany). Produced water samples were filtered through a 0.2 micron polyethersulfone membrane filter (Pall Corporation, Port Washington, NY, United States). The filter with the collected biomass was then transferred to a 1.5 mL microcentrifuge tube incubated with 1 mL TE buffer and 10 μl of 20 mg/mL lysozyme (Sigma Aldrich) at 37°C for 30 min. Samples were exposed to 10 min of bead beating on a vortexer followed by extraction using the recommended guidelines in the manufacturer’s protocol. DNA was eluted from spin filters in 100 μL nuclease free water. Four kit blanks were also concurrently extracted to confirm that no contamination occurred.

### Quantitative PCR

The bacterial abundance in produced water samples was measured using qPCR with 16S rRNA gene primers (F: GTGSTGCAYGGYTGTCGTCA; R: ACGTCRTCCMCACCTTCCTC) designed by Maeda et al. (2003) with an expected amplicon size of 146 base pairs. qPCR reactions were run in triplicate on a Magnetic Induction Cycler (MIC) (Bio Molecular Systems, Upper Coomera, Australia). Each reaction contained 2X SensiFAST SYBR No-Rox master mix (Bioline, London, United Kingdom), 400 nM forward primer, 400 nM reverse primer, and 1 μL of template DNA for a total reaction of 20 μL. qPCR conditions consisted of a polymerase activation step at 95°C for 2 min followed by 40 amplification cycles each consisting of: denaturation at 95°C for 5 s, annealing at 62°C for 5 s, and an extension step at 72°C for 1 s. Standard curves were generated using gBlocks Gene Fragments (Integrated DNA Technologies, Coralville, IA, United States) (Supplementary Data 1 and Supplementary Figure 1) and negative control samples were included in each amplification assay.

### 16S rRNA Library Preparation and Sequencing

Extracted DNA and kit blanks were amplified using universal primers targeting the V4 region of the 16S rRNA gene, as previously described (Caporaso et al., 2011, 2012). In order to obtain enough PCR product, we pooled 8 technical replicates per sample. The pooled PCR products were cleaned using AMPure or SPRIselect beads (Beckman Coulter, Pasadena, CA), quantified using the Qubit dsDNA High Sensitivity Assay Kit (Life Technologies, Carlsbad, CA, United States), and visualized on a Bioanalyzer using the High Sensitivity DNA Kit (Agilent, Santa Clara, CA, United States). Negative PCR controls were also amplified, quantified, and visualized in order to confirm that no contamination occurred. In total, we attempted to amplify 4 kit extraction blanks and 6 negative PCR controls. After quantification and visualization, only one amplified kit extraction blank and one negative PCR control contained amplicons. These samples, identified as Blank 1 and 2 in the Supplementary Data 1, were sequenced with our experimental 16S rRNA libraries. Purified 16S rRNA libraries were pooled, diluted to a concentration of 2 nM, and denatured using fresh 0.2 M NaOH. Libraries were further diluted according to the manufacturer’s instructions and sequenced on an Illumina Miseq (Illumina, San Diego, CA, United States) using a 300 cycle V2 Nano kit.

### 16S rRNA Data Analysis

16S rRNA gene sequences were analyzed using QIIME2 version 2019.10 (Bolyen et al., 2019). Sequences were imported as EMPSingleEndSequences and demultiplexed using the demux emp-single command. DADA2 (Callahan et al., 2016) was used to filter, denoise, and remove chimeras from the demultiplexed sequencing data. For this command, we utilized default settings and set the truncation length to 250 base pairs. The classify-sklearn (Pedregosa et al., 2011) command was used to classify representative sequences identified through DADA2 using a pre-trained Naive Bayes classifier trained on Silva 132 99% OTUs (Quast et al., 2012; Yilmaz et al., 2014) from the 515F/806R region and any sequences identified as chloroplast or mitochondria were filtered from the data.

Data generated by Qiime2 was imported into R for further analysis (R Core Team, 2013). We utilized the vegan package (Oksanen et al., 2012) to calculate the Chao1 index, Shannon index, and Bray–Curtis dissimilarity values. We also used the vegan package (Oksanen et al., 2012) to complete non-metric multidimensional scaling (NMDS), calculate Analysis of Similarities (ANOSIM), complete the Mantel test, and to fit environmental parameters onto the generated NMDS ordination plot. All NMDS plots were constructed with *k* = 3 dimensions. NMDS, ANOSIM, and the Mantel test were completed using Bray–Curtis distance measurements calculated after sequence libraries were resampled to the depth of the sample with the fewest sequences from this experiment (1942 sequences) (Supplementary Table 1). The Mantel test also relied on the Euclidean distance of the geochemical data, which was calculated in Base R. The significance of the ANOSIM analysis, the Mantel test, and the environmental vectors was based on 999 permutations of the grouping, geochemical, and environmental data, respectively. Finally, we also used the RColorBrewer package to generate colorblind accessible palettes when necessary (Neuwirth, 2014).

## Results

### Bakken Shale Exhibits High TDS Primarily Comprised of NaCl and a Localized Sulfate Trend

Produced water samples were obtained from 14 wells in the Bakken region (Figure 1). The pH was slightly below circumneutral with a range of 6.1–6.7 (Table 1), which is similar to previously reported results (Wang et al., 2019). The exception was well 6, which had a pH of 5.8 (Table 1). Alkalinity varied from low to high, with the majority of wells ranging from 186 to 740 mg/L as CaCO_3_. Exceptions were wells 5, 6, 7, and 10, which ranged from 1440 to 2000 mg/L as CaCO_3_ (Table 1). Although alkalinity of 2000 mg/L and higher have been reported in this region (Blondes et al., 2019; Chang et al., 2019), the variation is notable, as previous work involving these wells observed only low alkalinity measurements (Lipus et al., 2018). TOC was measured in the form of non-purgeable organic carbon (NPOC), which ranged from 63 to 543 mg as C/L (Table 1), and is similar to previously reported DOC values for the Bakken region (Wang et al., 2019).

**TABLE 1:** Well characteristics and produced water geochemical and microbial abundance measurements.

Well no.	Well age* (months)	Formation**	TDS (mg/L)	pH	Alkalinity (mg/L)	TIC (mg/L)	NPOC (mg/L)	16S rRNA gene copes/mL
1	52	BKN	215484	6.5	330	23.5	291	223
3	49	BKN	277081	6.3	500	29.8	76	83
4	49	TF1	229002	6.5	450	47.2	139	149
5	79	BKN	330664	6.3	1440	13.2	116	102
6	79	TF1	326055	5.8	2000	20.9	140	66
7	49	BKN	343563	6.1	1600	12.3	342	63
8	49	BKN	354667	6.1	740	11.7	63	180
10	61	BKN	294780	6.4	1750	16.4	407	65
11	55	BKN	134561	6.9	440	38.2	543	305800
12	55	TF1	303176	6.7	560	13.8	71	22
13	55	TF2	311195	6.4	398	12.2	90	404
14	55	BKN	270392	6.8	426	19.1	333	325
17	53	TF1	356018	6.4	186	4.1	66	127
18	1	U	290236	6.4	310	24.8	326	30

*Well age at May 2018 sampling event. **BKN, Bakken formation; TF1, First bench of Three Forks formation; TF2, Second bench of Three Forks formation; U, Unavailable Data.

**FIGURE 2:**
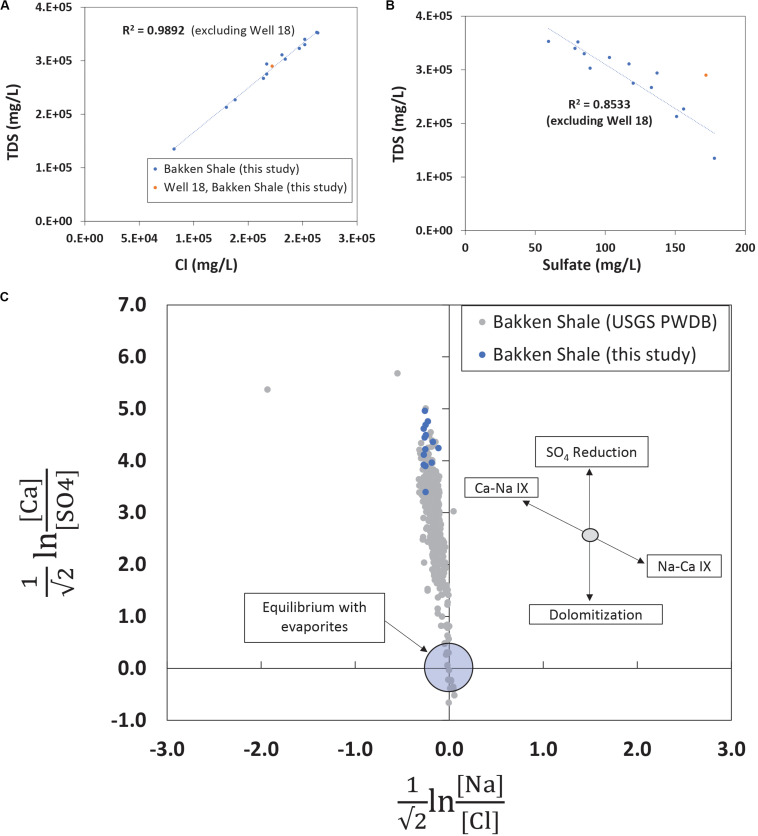
**(A)** TDS and chloride measurements plotted for produced waters in this study. Linear trendlines report a high linear correlation value, displaying that chloride is the primary anion and well 11, with its lower TDS value, does not have an anomalous chloride value. The trendline excludes well 18, a sample that, based on its geochemistry and age, likely represents a mix of flowback and produced water. **(B)** TDS and sulfate measurements plotted for produced waters in this study. There is a higher linear association (*R*^2^ = 0.8533) for these analytes when the youngest and spatially unique sample, well 18, is excluded. Based on measurements from this study and previous research, the elevated sulfate in well 18 could be related to residual chemicals from hydraulic fracturing operations or the product of flowback waters. **(C)** Isometric log-ratios for produced waters from the Bakken Shale. Data collected with this study are shown in blue while all data available from the Bakken region in the USGS PWDB (Blondes et al., 2019) is shown in gray. This figure portion is modified from Engle et al. (2016).

The TDS concentrations ranged between 135,000 mg/L and 357,000 mg/L (Table 1) and were characterized by 86.1% wt to 88.6% wt Na^+^ and Cl^–^ (Table 2). These TDS concentrations are similar to those previously reported within the Bakken Shale (Thyne and Brady, 2016; Lipus et al., 2018; Wang et al., 2019) and reinforce that produced water from the Bakken region has higher TDS concentrations than samples collected from other shale plays, including the Marcellus and Barnett (Akob et al., 2015; Lipus et al., 2017; Wang et al., 2019). These high salinities are likely derived from post-depositional halite and anhydrite dissolution processes (Bachu and Hitchon, 1996; Iampen and Rostron, 2000). TDS measurements in conjunction with major cation and anion concentrations can either reflect the formation brine (Iampen and Rostron, 2000; Engle et al., 2016) or fluid mixing processes related to hydraulic fracturing at unconventional wells (Haluszczak et al., 2013; Vengosh et al., 2017). Samples in this study display a positive linear association (*R*^2^ = 0.9892) between TDS and chloride (Figure 2A), a trend that is seen in other Bakken produced waters (Thyne and Brady, 2016) and is expected since chloride is the primary anion in this study’s samples. This trendline includes well 11, suggesting that this water’s TDS and chloride concentrations are still compositionally similar to other samples in this study. This is notable, as produced water from well 11 exhibited a lower TDS concentration (135,000 mg/L) compared to the other samples, which had an average of 287,000 mg/L (Table 1). TDS levels from well 11 also fall within the range of recorded Bakken produced water measurements when data reported in the USGS PWDB were plotted along with samples from this study (Blondes et al., 2019) (Supplementary Figure 2A). The low TDS of well 11 may be due to its spatial heterogeneities within the reservoir; Bakken produced waters have high spatial TDS variability, as there is minimal fluid flow in certain regions due to the formation’s aquitard characteristics (Bachu and Hitchon, 1996; Hitchon, 1996).

**TABLE 2:** Major cation and anion concentrations with selected trace metal concentrations for produced waters.

Well no.	Ba	Br	Ca	Cl	Fe^#^	K	Mg	Mn^#^	Na	P^#^	Sr	SO_4_
1	53.1	656	16100	130000	179.1	5770	912	19.6	57400	12.0	2200	151
3	66.8	854	19600	167000	161.8	7470	1120	75.0	75700	1.47	2690	120
4	57.3	711	16100	138000	229.6	6380	883	17.3	62500	3.12	2160	156
5	31.3	1050	24300	202000	201.9	8900	1350	151	89200	1.60	3160	85.0
6	76.2	1050	23200	197000	151.3	8940	1230	147	88200	3.27	3000	103
7	72.1	1020	27300	202000	206.5	9110	1510	110	94900	11.0	3710	78.4
8	107.0	1020	25500	214000	192.3	9210	1390	114	97300	1.07	2650	80.5
10	BDL	817	23100	167000	96.8	6830	1520	22.9	91900	6.22	2870	137
11	12.0	406	9080	81900	158.0	3860	709	8.49	37300	1.43	1060	178
12	BDL	1090	21400	184000	374.8	8690	1230	27.1	83900	0.69	2640	89.2
13	20.0	1100	23300	181000	250.2	9440	1390	20.3	92000	BDL	2560	117
14	BDL	1140	18700	164000	162.8	6990	1370	33.5	72600	2.20	2360	133
17	63.7	1160	27700	213000	209.4	9660	1640	61.4	95800	0.273	3500	59.5
18	BDL	913	19500	172000	63.1	7660	1240	12.5	85700	BDL	2540	172

*All measurements are reported in mg/L and were taken using IC unless otherwise noted. ^#^Measured using ICP-OES.*

Sulfate is a typical secondary anion in produced waters and increases in sulfate concentration can reflect drilling activity, either due to injected chemicals associated with hydraulic fracturing operations (Paukert Vankeuren et al., 2017) or due to injected water reacting with sulfur and sulfate bearing minerals (Osselin et al., 2019). Although anhydrite content is negligible, trace amounts of pyrite have been observed in the Bakken Formation (Pitman et al., 2001; Ashu, 2014). When TDS is plotted against sulfate and the youngest and most spatially unique sample (well 18) is excluded there is a negative linear relationship (*R*^2^ = 0.853) (Figure 2B). Although most wells were hydraulically fractured more than 4 years ago (Table 1), well 18 is 1 month removed from a completed hydraulic fracturing procedure. Therefore, the elevated sulfate concentration in well 18 is either the result of spatial heterogeneity or recent hydraulic fracturing. When TDS and sulfate for other Bakken produced waters are plotted (Supplementary Figure 2B), there is no clear linear association (*R*^2^ = 0.0811) between the parameters. This suggests samples from this study are exhibiting a localized trend. Despite this, the young age of well 18 makes it difficult to conclude whether it falls off the linear trendline (Figure 2B) due to its location or the recent hydraulic fracturing operation.

Previous research has demonstrated that evaluating produced water chemistry using compositional data analysis can remove inaccurate correlations (Engle and Rowan, 2013; Engle and Blondes, 2014; Blondes et al., 2016). When they are applied to produced waters, compositional data analysis techniques include using isometric log-ratio transformations to understand whether concentration variations indicate water-rock or fluid mixing processes. Molar ratios of four analytes (calcium, sulfate, sodium, and chloride) were transformed using isometric log-ratios and were plotted (Figure 2C) for Bakken produced waters. The region near the origin represents equilibrium conditions with the evaporite minerals halite and sulfate. Samples from this study plotted within the range of USGS PWDB samples, but they plotted on the high end of the calcium-sulfate ratios (Figure 2C). Previous work studying produced waters has suggested similarly elevated calcium-sulfate ratios may be the result of sulfate reduction (Engle et al., 2016). This suggests that sulfate reduction was more prevalent in samples from this study than other samples from the Bakken region.

### Produced Waters Contain Low Microbial Loads With High Alpha Diversity

In order to determine the total microbial abundance in the evaluated Bakken region produced waters, qPCR analyses were conducted on the 14 collected samples. The median microbial load was found to be 1.15E+2 16S rRNA gene copies/mL of produced water. The microbial abundances across all produced water samples ranged from 2.15E+1 to 3.06E+5 16S rRNA gene copies/mL. The microbial load in well 11 was significantly higher than in the other samples and appeared to be an outlier (Table 1 and Supplementary Figure 3). However, it falls within the microbial loads recorded in the Marcellus Shale and Denver-Julesburg Basin, which range from 1.00E+5 to 2.10E+8 and 1.0E+3.1 to 1.0E+8.5, respectively (Lipus et al., 2017; Hull et al., 2018).

To assess the microbial community composition, we conducted 16S rRNA gene amplicon sequencing for 11 of the 14 produced water samples. No DNA could be amplified for the remaining three samples, potentially due to low overall biomass. Additionally, 10 blanks were prepared, two of which had measurable DNA and were therefore sequenced. 16S rRNA amplicon sequencing yielded a total of 675,581 raw sequences, of which 289,457 remained after quality control filtering (Supplementary Table 1). We also concurrently analyzed previously published sequences (Lipus et al., 2018) generated from samples collected at the same sites and within the same region using the same bioinformatic pipeline (Supplementary Table 1).

Alpha diversity was determined by counting the total number of ASVs in a sample and calculating the Shannon richness, Chao1 index, and Pielou’s evenness metric for each site (Table 3, Supplementary Table 1, and Supplementary Figure 4). Each sample had an average of 58 unique ASVs per 1942 sequences. Chao1 indices ranged from 37 to 103, Shannon indices ranged from 2.42 to 3.68, and Pielou’s evenness measurements ranged from 0.64 to 0.89 per 1942 sequences across all produced water samples (Table 3, Supplementary Table 1, and Supplementary Figure 4). As a whole, our samples were significantly more rich, diverse, and even when compared to samples previously collected from the same wells by Lipus et al. (2018) (Supplementary Table 1 and Supplementary Figure 4). Our samples also had a greater spread of Chao1 indices and a smaller spread of both Shannon indices and Pielou’s evenness measurements (Supplementary Figure 4). The notable exception is that samples collected in January 2015 by Lipus et al. (2018) had comparable levels of evenness and a much smaller spread for all alpha diversity metrics (Supplementary Figure 4). One possible reason for this trend is that after several years of production, well conditions select for a microbial community with a high diversity of adapted microorganisms.

**TABLE 3:** Alpha diversity metrics^A^ for produced water samples.

Well no.	ASVs	Richness^R^	Diversity^D^	Evenness^E^
3	79	84.14	3.23	0.74
4	37	37.00	2.86	0.79
5	52	67.00	3.15	0.80
7	64	64.00	3.68	0.89
8	69	70.20	3.68	0.87
10	47	47.14	2.46	0.64
11	43	46.00	2.42	0.64
11	43	46.00	2.42	0.64
12	44	44.00	2.95	0.78
13	80	103.08	2.78	0.64
17	46	46.00	3.18	0.83
18	95	98.11	3.59	0.79

*^*A*^Metrics were calculated after sequence libraries were resampled to the depth of the sample with the fewest sequences from this experiment (1942 sequences), ^*R*^Chao1 Index, ^*D*^Shannon Index, ^*E*^Pielou’s Evenness.*

### Hydrocarbon Associated Bacterial Families Dominate Microbial Community

**FIGURE 3:**
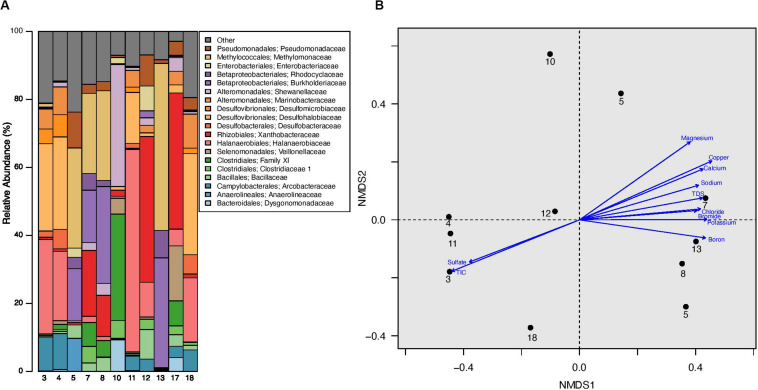
**(A)** Relative abundance of microbial families in produced water samples obtained from well separators. All families that represent = 5% of sequences from any one sample are listed in the barplot, all other families are grouped together under “Other.” **(B)** Non-metric multidimensional scaling (NMDS) plot of produced water samples with a stress value of 0.0720. This plot was constructed using Bray-Curtis distance calculated after sequence libraries were resampled to the depth of the sample with the fewest sequences from this experiment (1942 sequences). There was no significant clustering by well formation or well age (ANOSIM *p* > 0.05 for both), however, a Mantel test demonstrated a significant correlation between the microbial community and associated geochemical profile (*R* = 0.2336; *p* = 0.0430). Environmental vectors for geochemical parameters were fit onto the ordination plot, with the direction of the arrow corresponding to the direction of the gradient and the length of the vector proportional to the strength of the correlation between ordination and environmental variable. Only environmental vectors with a significant *p*-value (*p* < 0.05) are displayed; an NMDS plot with all environmental vectors as well as a table containing the *R*^2^ and *p*-values for the corresponding vectors is located in Supplementary Figure 6. In this figure, TIC refers to Total Inorganic Carbon (TIC).

The analyzed produced water samples were dominated by microbiota from 19 bacterial families, each of which represents ≥ 5% of sequences from any one sample. Twelve of the dominant families have been previously isolated and cultured from hydrocarbon reservoirs and 5 have been previously identified through sequencing hydrocarbon-associated environmental samples (Korenblum et al., 2012; Tatar, 2018). These dominant families comprise between 76.24% and 97.08% of the total microbial community in each well (Figure 3A, Supplementary Table 3, and Supplementary Figure 5). The families Halanaerobiaceae, Methylomonaceae, Xanthobacteraceae, Burkholderiaceae, Desulfohalobiaceae, and Pseudomonadaceae were especially abundant, with an average relative abundance of 57.02% and a range between 4.43 and 83.24% (Figure 3A, Supplementary Table 2, and Supplementary Figure 5). The one exception is produced water from well 10, which was dominated by the Clostridiales Family XI and Shewanellaceae, representing 35.80 and 31.32%, respectively, of the total microbial community (Figure 3A, Supplementary Table 3, and Supplementary Figure 5). Shewanellaceae has previously been detected in saline environments, oil field wastes, and other hydrocarbon environments (Nealson and Scott, 2006), suggesting an adaptability to conditions similar to unconventional reservoirs. It was previously found in produced water from the Bakken region (Lipus et al., 2018), although only at low abundances, suggesting adaptation to well conditions over time. No other wells contained such high abundances of Family XI and Shewanellaceae. However, Family XI and Shewanellaceae were present in the remaining produced water samples with an average relative abundance of 2.17 and 1.58%, respectively (Figure 3A, Supplementary Table 3, and Supplementary Figure 5).

Two other identified bacterial families include Desulfobacteraceae and Desulfomicrobiaceae, which have an average relative abundance of 1.13 and 1.09% across all produced water samples, respectively (Figure 3A, Supplementary Table 3, and Supplementary Figure 5). Both families were especially dominant in well 4, constituting 5.72 and 6.54%, respectively, of the total relative abundance (Figure 3A, Supplementary Table 3, and Supplementary Figure 5). Desulfobacteraceae also represented 5.6% of the relative abundance in well 18 (Figure 3A, Supplementary Table 3, and Supplementary Figure 5). Desulfobacteraceae and Desulfomicrobiaceae are putative sulfate reducers and may directly contribute to well souring, corrosion, and/or other operational failures. Bacillaceae were found in every produced water sample and accounted for 0.01–8.67% of the total community within each well (Figure 3A, Supplementary Table 3, and Supplementary Figure 5). This is especially notable as samples previously collected at the same sites were dominated by Bacillales (Supplementary Figure 5) (Lipus et al., 2018). Finally, Veillonellaceae (Order: Selenomonadales) were detected in 7 of the 11 produced water samples and constituted 16.17% of the microbiota present in well 17 (Figure 3A, Supplementary Table 3, and Supplementary Figure 5). The remaining dominant bacterial families include Dysgonomonadaceae, Marinobacteraceae, Anaerolineaceae, Rhodocyclaceae, Arcobacteraceae, Clostridiaceae 1, and Enterobacteriaceae (Figure 3A, Supplementary Table 2, and Supplementary Figure 5). None of these taxa were found in every produced water sample and they were identified at average relative abundances of 0.88–3.50% across all wells. Archaea were also present in six of the 11 produced water samples and contributed a maximum of 0.89% of the relative abundance in any one well. The archaeal orders present in our samples included Methanobacteriales and Methanomicrobiales, which are common methanogens previously implicated in hydrocarbon degradation (Jiménez et al., 2016; Toth and Gieg, 2018).

### Microbial Community Correlates With Geochemical Data

We completed several analyses in order to identify factors potentially contributing to and/or driving changes in the produced water microbial ecology. First, we constructed three non-metric multidimensional scaling (NMDS) plots using Bray-Curtis dissimilarity metrics. These ordination plots contain: (1) all samples and blanks from our current study (Supplementary Figure 6A), (2) samples and blanks from our current study as well as samples previously collected by Lipus et al. (2018) from the same sample sites (Supplementary Figure 6B), and (3) samples and blanks from our current study and all samples previously collected by Lipus et al. (2018) from the Bakken region (Supplementary Figure 6C). Next, we used analysis of similarity (ANOSIM) calculations to confirm any visible clustering present in each of the three plots. We specifically tested for the clustering by the following categories, when possible: sample origin (tank, separator, or blank), well number, formation, well age, and sampling event. We found no statistically significant clustering based on sample origin, formation, or well age (ANOSIM: *p* > 0.05) in our first plot (Supplementary Figure 6A). However, incorporation of previously collected data at the sample sample sites revealed clustering by sample origin (ANOSIM: *R* = 0.1769; *p* < 0.05), well age (ANOSIM: *R* = 0.4350; *p* < 0.05), and sampling event (ANOSIM: *R* = 0.5622; *p* < 0.05). Samples did not cluster by well number or formation (ANOSIM: *p* > 0.05 for both). Interestingly, when we completed an ANOSIM with our experimental data and the paired 2018 data (but without our sequenced blanks), there was no separation by sample origin (ANOSIM: *p* > 0.05). These trends remained consistent when we analyzed our sequenced blanks, experimental data, and all previously published sequences by Lipus et al. (2018) (Supplementary Figure 6C). We found no significant clustering by sample origin, well number, or formation although there was significant clustering by well age (ANOSIM: *R* = 0.4533; *p* = 0.001) and sampling event (ANOSIM: *R* = 0.4874; *p* = 0.001) (Supplementary Figure 6C).

In order to examine the relationship between the microbial community within each well and the associated geochemical profile, we completed a Mantel test. We found a statistically significant, but weak relationship (*R* = 0.2336; *p* = 0.0430). Next, we calculated and fit vectors onto a fourth ordination plot, which contained only our experimental data, in order to identify environmental factors associated with produced water microbial structure (Figure 3B and Supplementary Figure 7). We determined that 11 of the 29 geochemical measurements have a significant correlation with the microbial data visualized on the NMDS plot (Figure 3B and Supplementary Figure 7). The *R*^2^ of the significant correlations was strong, ranging from 0.56 to 0.84 with an average *R*^2^ of 0.65 (Supplementary Figure 7). TDS was significantly correlated with the microbial data, with an *R*^2^ of 0.62. TDS is a measurement of the total amount of minerals, salts, metals, cations, and/or anions dissolved in water, therefore it is unsurprising that many of the TDS constituents also had a significant correlation with the microbial data. However, there were several notable trends. First, the Bakken Shale is known to have high NaCl concentrations compared to other shale regions (Table 2 and Supplementary Table 2) (Lipus et al., 2018). Consistent with this, sodium and chloride were significantly correlated with the microbial data (*R*^2^ = 0.62 and *R*^2^ = 0.59, respectively), although NaCl% was not. Second, the *R*^2^ for boron, calcium, copper, potassium, and manganese were higher than the R2 for TDS, suggesting that these ions may be especially linked to microbial metabolism within the Bakken Shale Formation. Other geochemical measurements with a significant correlation to the microbial data include bromide, sulfate, and TIC (Figure 3B and Supplementary Figure 7).

## Discussion

To contribute to the expanding portfolio of produced water descriptions in the United States and build on previous work conducted in the Bakken Shale, we analyzed produced water samples from 14 different wells using integrated geochemical and microbiology techniques. Previous work demonstrates produced water from the Bakken region contains high TDS and salinity concentrations compared to other shale regions (Lipus et al., 2018; Wang et al., 2019). Our work supports this, with samples containing an average of 287,000 mg/L TDS and 88% wt NaCl across all wells (Table 1). These conditions inhibit microbial growth, such that produced water generally exhibits low microbial loads. Previous work in the Bakken Shale reported microbial abundances of 1.00E+2 to 1.00E+5 16S rRNA gene copies/mL (Lipus et al., 2018). Consistent with this, we measured a median microbial load of 1.15E+2 16S rRNA gene copies/mL across all samples. The highest microbial abundance was recorded for produced waters from well 11 at 3.06E+5 16S rRNA gene copies/mL (Table 1 and Supplementary Figure 3). Although the microbial load in produced water from well 11 was higher than in the other collected samples within this dataset, the well age and geochemical characteristics, including major anion concentrations and isometric log-ratios, suggest that this water is representative of Bakken produced waters and that its low TDS is not the result of dilution due to hydraulic fracturing operations (Figure 2 and Supplementary Figure 2A). Rather, it is likely that its low TDS is due to the aquitard nature of the Bakken formation, which results in high spatial TDS variability (Bachu and Hitchon, 1996; Hitchon, 1996).

In addition to inhibiting microbial growth, the unique conditions in shale reservoirs also select for organisms that thrive in anaerobic, halophilic conditions. Four previous studies have explicitly examined the Bakken Shale microbial community *in situ* (Strong et al., 2014; An et al., 2017; Lipus et al., 2018; Wang et al., 2019). Strong et al. (2014) found a high abundance of *Halanaerobium* and *Marinobacter* in flowback water; An et al. (2017) discovered high abundances of *Marinobacter*, *Halanerobium*, and *Desulfovermiculus* in produced water samples; Lipus et al. (2018) found that produced samples were dominated by microbes in the Bacillales, Halanaerobiales, or Pseudomonadales orders*;* and Wang et al. (2019) reported a high abundance of *Geobacter, Lactococcus, Enterococcus*, and *Bradyrhizobium* in wastewater from newly fractured wells. Despite a significant difference in the structure of the microbial communities, our results were broadly congruent with those presented in these studies. Three of our dominant bacterial families belonged to the Bacillales, Halanaerobiales, and Pseudomonadales orders. Across all wells, *Halanaerobium* represented an average of 99.58% of the bacteria within the Halanaerobiales order. Additionally, *Marinobacter* and *Bradyrhizobium* were especially prevalent, representing an average of 56.59 and 95.26% of all microbiota in the Alteromonadales and Rhizobiales orders, respectively. It is unknown whether the microbial communities in the Bakken region in this study and other studies were native to the reservoir, or whether they were “seeded” or inoculated during well development and normal operating procedures. Regardless, the geochemical characteristics were within range of the Bakken region, strongly suggesting a long-term adaptation of the community, as opposed to a temporary augmentation to the system.

Our samples were characterized by high alpha diversity and evenness compared to the other Bakken studies. They also contained high abundances of other taxa including members of the Methylomonaceae, Desulfobacteraceae, and Desulfomicrobiaceae families. Although these families have previously been detected in produced water, their unusually high abundances and the overall high evenness of these samples is notable. One hypothesis of the unusually high evenness is that the advanced age of these wells has selected for a more stable, even community.

We did not recover enough DNA for metagenomic analysis, however, previous studies of the abundant microorganisms shed some light in potential metabolic processes that may impact the oil and gas industry. For example, metagenome-assembled genomes (MAGs) of Halanaerobium recovered from the Marcellus and Utica Shales and a Halanaerobium strain isolated from the Barnett Shale were found to be capable of thiosulfate reduction (Daly et al., 2016; Liang et al., 2016; Lipus et al., 2017). Thiosulfate reduction is highly linked with microbially induced corrosion (MIC) (Choudhary et al., 2015) and sulfide production (Booker et al., 2017), which can damage well infrastructure and cause reservoir souring. Desulfobacteraceae and Desulfomicrobiaceae are also well known sulfate reducers that have previously been implicated in reservoir souring (Gieg et al., 2011; Engelbrektson et al., 2014; Vigneron et al., 2017). The Halanaerobium MAGs recovered from unconventional reservoirs contained genes encoding proteins involved with the biofilm formation process (Daly et al., 2016; Lipus et al., 2017). Microbial biofilms are a major issue in the oil and gas industry, as they can lead to clogging and increase the rates and/or severity of MIC (Johnson et al., 2008; Bottero et al., 2010; Struchtemeyer et al., 2012). *Pseudomonas* are especially well known due to their biofilm formation capabilities (Drenkard and Ausubel, 2002; Vikram et al., 2015) and have been shown to increase biofilm production when exposed to produced water (Vikram et al., 2014). *Pseudomonas* composed the majority of the sequences from the Pseudomonadles order in our samples (79.93% across all wells) except for well 4, which contained no *Pseudomonas*.

One of our goals was to understand the complicated geochemical and microbial dynamics present within the Bakken shale. We determined that 11 of the 29 geochemical measurements have a significant correlation with the microbial data (Supplementary Figure 6). These include TDS and many of its constituents such as sodium and chloride concentrations, which are higher in the Bakken region than in other producing shale formations (Lipus et al., 2018; Wang et al., 2019). This suggests these parameters shape the microbial ecology of Bakken Shale produced water, and these variables have an expected positive trend with TDS. Sulfate concentration also had a significant correlation with the microbial data, although our geochemical analysis revealed a negative trend between TDS and sulfate. One possible reason for this may be that certain sulfate minerals, specifically gypsum and anhydrite, are less soluble in higher TDS waters (Bock, 1961; Tanveer and Chen, 2020). This theory would suggest that low TDS wells, such as well 11, would allow for more sulfate to remain in solution; this low TDS, high sulfate niche may provide a unique environment for microbial activity. More research is needed to determine potential sulfate solubility and related microbial activity in unconventional reservoirs.

The isometric log-ratio plot showed that produced water samples from this study plotted within the range of USGS PWDB samples, but they plotted on the high end of the calcium-sulfate ratios (Figure 2C). This suggests that sulfate reduction was more prevalent in samples from this study than other samples collected from the Bakken region. This sulfate reduction could have occurred at any point during the geologic life of this produced water. However, previous research has detected the presence of sulfate reducing bacteria in Bakken produced waters, specifically in those with lower salinities (An et al., 2017). This is consistent with our observations in well 11, which has a low TDS, a high sulfate concentration, and high microbial load that is dominated by sulfate reducers. The majority of these sulfate reducers (14.96%) belong to the *Desulfovermiculus* genera, which was previously detected in the Bakken region by An et al. (2017). Members of this taxon have also been isolated from hydrocarbon associated environments, and shown to be chemolithoautotrophic and halophilic (Belyakova et al., 2006). Closely related organisms in the Desulfovibrionales order have also previously been associated with both MIC and reservoir souring (Gieg et al., 2011; Chen et al., 2015).

Produced water from well 11 and 18 both contain elevated sulfate concentrations, however, only well 11 had a high microbial load. This is most likely due to the high TDS concentration present in the produced water from well 18, which inhibits microbial growth. Given this context, it is possible that the lower salinity in combination with high sulfate concentrations resulted in the high microbial abundance observed in well 11, and suggests that sulfate reduction may have been actively occurring in this sample. Since the chloride and TDS trends suggest well 11 to be in range of other Bakken wells, this further suggests that the Bakken Shale may host heterogenous niches with conditions that are conducive to microbial growth and activity. Although our experimental data for well 18 does not support active microbial sulfate reduction, it is important to understand that the Bakken Shale is a dynamic ecosystem. First, consider that well 18 is 1 month removed from a completed hydraulic fracturing procedure, thus any new microbes introduced during the fracturing process may still be below the detection limit. Additionally, previous work demonstrates that the shale-associated microbial community shifts in response to changing geochemical conditions in the months after oil and gas production begins (Daly et al., 2016). Finally, when we analyzed our data concurrently with data previously collected within the same region (Lipus et al., 2018), we found a significant difference in the microbial community that correlated with well age and sample event. This suggests microbial community divergence at well age orders of years as opposed to shorter termed time periods.

This work represents the first study to couple geochemical trend analysis with microbial data for oil and gas systems, demonstrating the geochemical parameters have the potential to shape the microbial community present in the Bakken Shale. Our integrated approach provides valuable context to the three previously completed studies in this region, however, additional work must be completed in order to understand the complex dynamics present in hydraulically fractured wells. For example, our results demonstrate the microbial community can change within the same wells after a year of production; additional 16S rRNA gene sequencing in this region would allow us to better determine whether seasonality, well age, or another unidentified environmental factor is the primary driver of microbial community in the Bakken Shale. Utilizing metagenomic and/or metatranscriptomic techniques within this region will help us identify mechanistic links between the produced water microbiota and chemistry. Finally, a meta-analysis of all hydraulically fractured well chemical and microbial data may help us identify broader trends present in all unconventional reservoirs. Ultimately, our goal is to better understand the microbiology and geochemistry of hydraulically fractured wells so we can continue to safely and efficiently utilize our natural resources.

## Data Availability Statement

The datasets presented in this study can be found in online repositories. The names of the repository/repositories and accession number(s) can be found at: https://www.ncbi.nlm.nih.gov/, PRJNA611830.

## Author Contributions

KT filtered the samples for microbial analysis, prepared and sequenced the 16S rRNA gene sequencing libraries, analyzed the 16S rRNA gene sequencing data, and wrote the manuscript. JG analyzed the geochemical data and wrote the manuscript. DL collected the field samples, recorded the field data, and preserved the field samples. PS filtered the samples for microbial analysis, collected the qPCR data, and prepared and sequenced the 16S rRNA gene sequencing libraries. MS collected the ICP-OES and IC measurements. DG procured the funding, collected the field samples, recorded the field data, preserved the field samples, and analyzed the geochemical data and qPCR data. All the authors reviewed and provided editorial feedback.

## Disclaimer

This presentation was prepared as an account of work sponsored by an agency of the United States Government. Neither the United States Government nor any agency thereof, nor any of their employees, makes any warranty, express or implied, or assumes any legal liability or responsibility for the accuracy, completeness, or usefulness of any information, apparatus, product, or process disclosed, or represents that its use would not infringe privately owned rights. Reference therein to any specific commercial product, process, or service by trade name, trademark, manufacturer, or otherwise does not necessarily constitute or imply its endorsement, recommendation, or favoring by the United States Government or any agency thereof. The views and opinions of authors expressed therein do not necessarily state or reflect those of the United States Government or any agency thereof.

## Conflict of Interest

The authors declare that the research was conducted in the absence of any commercial or financial relationships that could be construed as a potential conflict of interest.

## References

[B1] Akob D. M. Cozzarelli I. M. Dunlap D. S. Rowan E. L. Lorah M. M. (2015). Organic and inorganic composition and microbiology of produced waters from Pennsylvania shale gas wells. *Appl. Geochem.* 60 116–125. 10.1016/j.apgeochem.2015.04.011

[B2] Akyon B. McLaughlin M. Hernández F. Blotevogel J. Bibby K. (2019). Characterization and biological removal of organic compounds from hydraulic fracturing produced water. *Environ. Sci.* 21 279–290. 10.1039/C8EM00354H 30451271

[B3] An B. A. Shen Y. Voordouw G. (2017). Control of sulfide production in high salinity bakken shale oil reservoirs by halophilic bacteria reducing nitrate to nitrite. *Front. Microbiol.* 8:1164. 10.3389/fmicb.2017.01164 28680423 PMC5478722

[B4] Arthur J. D. Bohm B. Coughlin B. J. (2008). *Hydraulic Fracturing Considerations For Natural Gas Wells Of The Marcellus Shale.* Tulsa, OK: ALL Consulting, LLC.

[B5] Ashu R. (2014). A petrographic study of the three forks formation (upper devonian), williston basin, north dakota: based on thin section analysis, XRD and SEM. *J. Geol. Res.* 2014 1–9. 10.1155/2014/264170

[B6] Bachu S. Hitchon B. (1996). *Regional-Scale Flow Of Formation Waters In The Williston Basin. AAPG Bulletin 80.* Available online at: https://www.osti.gov/biblio/232993 (accessed March 30, 2020).

[B7] Barbot E. Vidic N. S. Gregory K. B. Vidic R. D. (2013). Spatial and temporal correlation of water quality parameters of produced waters from devonian-age shale following hydraulic fracturing. *Environ. Sci. Technol.* 47 2562–2569. 10.1021/es304638h 23425120

[B8] Belyakova E. V. Rozanova E. P. Borzenkov I. A. Tourova T. P. Pusheva M. A. Lysenko A. M. (2006). The new facultatively chemolithoautotrophic, moderately halophilic, sulfate-reducing bacterium *Desulfovermiculus halophilus* gen. nov., sp. nov., isolated from an oil field. *Microbiology* 75 161–171. 10.1134/S0026261706020093 16758868

[B9] Blondes M. S. Engle M. A. Geboy N. J. (2016). “A practical guide to the use of major elements, trace elements, and isotopes in compositional data analysis: applications for deep formation brine geochemistry,” in *Compositional Data Analysis Springer Proceedings in Mathematics & Statistics*, eds Martín-Fernández J. A. Thió-Henestrosa S. (Cham: Springer International Publishing), 13–29. 10.1007/978-3-319-44811-4_2

[B10] Blondes M. S. Gans K. D. Engle M. A. Kharaka Y. K. Reidy M. E. Saraswathula V. (2019). *U.S. Geological Survey National Produced Waters Geochemical Database ver. 2.3, May 2019.* Reston, VA.: U.S. Geological Survey.

[B11] Bock E. (1961). On the solubility of anhydrous calcium sulphate and of gypsum in concentrated solutions of sodium chloride at 25 °C, 30 °C, 40 °C, and 50 °C. *Can. J. Chem.* 39 1746–1751. 10.1139/v61-228

[B12] Bolyen E. Rideout J. R. Dillon M. R. Bokulich N. A. Abnet C. C. Al-Ghalith G. A. (2019). Reproducible, interactive, scalable and extensible microbiome data science using QIIME 2. *Nat. Biotechnol.* 37 852–857. 10.1038/s41587-019-0209-9 31341288 PMC7015180

[B13] Booker A. E. Borton M. A. Daly R. A. Welch S. A. Nicora C. D. Hoyt D. W. (2017). Sulfide generation by dominant halanaerobium microorganisms in hydraulically fractured shales. *mSphere* 2:e00257-17. 10.1128/mSphereDirect.00257-17 28685163 PMC5497025

[B14] Bottero S. Picioreanu C. Enzien M. V. Van Loosdrecht M. Bruining J. Heimovaara T. (2010). “Formation damage and impact on gas flow caused by biofilms growing within proppant packing used in hydraulic fracturing,” in *Proceedings of the SPE International Symposium and Exhibition on Formation Damage Control* (Lafayette, LA: Society of Petroleum Engineers). 10.2118/128066-MS

[B15] Callahan B. J. McMurdie P. J. Rosen M. J. Han A. W. Johnson A. J. A. Holmes S. P. (2016). DADA2: high-resolution sample inference from Illumina amplicon data. *Nat. Methods* 13 581–583. 10.1038/nmeth.3869 27214047 PMC4927377

[B16] Caporaso J. G. Lauber C. L. Walters W. A. Berg-Lyons D. Huntley J. Fierer N. (2012). Ultra-high-throughput microbial community analysis on the Illumina HiSeq and MiSeq platforms. *ISME J.* 6 1621–1624. 10.1038/ismej.2012.8 22402401 PMC3400413

[B17] Caporaso J. G. Lauber C. L. Walters W. A. Berg-Lyons D. Lozupone C. A. Turnbaugh P. J. (2011). Global patterns of 16S rRNA diversity at a depth of millions of sequences per sample. *Proc. Natl. Acad. Sci. U.S.A.* 108 4516–4522. 10.1073/pnas.1000080107 20534432 PMC3063599

[B18] Chang H. Li T. Liu B. Vidic R. D. Elimelech M. Crittenden J. C. (2019). Potential and implemented membrane-based technologies for the treatment and reuse of flowback and produced water from shale gas and oil plays: a review. *Desalination* 455 34–57. 10.1016/j.desal.2019.01.001

[B19] Chen Y. Tang Q. Senko J. M. Cheng G. Zhang Newby B. Castaneda H. (2015). Long-term survival of *Desulfovibrio vulgaris* on carbon steel and associated pitting corrosion. *Corros. Sci.* 90 89–100. 10.1016/j.corsci.2014.09.016

[B20] Choudhary L. Macdonald D. D. Alfantazi A. (2015). Role of thiosulfate in the corrosion of steels: a review. *CORROSION* 71 1147–1168. 10.5006/1709

[B21] Cluff M. A. Hartsock A. MacRae J. D. Carter K. Mouser P. J. (2014). Temporal changes in microbial ecology and geochemistry in produced water from hydraulically fractured Marcellus shale gas wells. *Environ. Sci. Technol.* 48 6508–6517. 10.1021/es501173p 24803059

[B22] Daly R. A. Borton M. A. Wilkins M. J. Hoyt D. W. Kountz D. J. Wolfe R. A. (2016). Microbial metabolisms in a 2.5-km-deep ecosystem created by hydraulic fracturing in shales. *Nat. Microbiol.* 1:16146. 10.1038/nmicrobiol.2016.146 27595198

[B23] Davis J. P. Struchtemeyer C. G. Elshahed M. S. (2012). Bacterial communities associated with production facilities of two newly drilled thermogenic natural gas wells in the Barnett shale (Texas, USA). *Microb. Ecol.* 64 942–954. 10.1007/s00248-012-0073-3 22622766

[B24] Drenkard E. Ausubel F. M. (2002). *Pseudomonas* biofilm formation and antibiotic resistance are linked to phenotypic variation. *Nature* 416 740–743. 10.1038/416740a 11961556

[B25] Elliott A. S. Suri N. K. An D. Voordouw G. Kaert K. Langille D. L. (2014). “Analysis of microbes in hydraulic fracturing of montney tight gas formations in Western Canada,” in *Proceedings of the SPE/CSUR Unconventional Resources Conference - Canada*, Calgary, AB.

[B26] Engelbrektson A. Hubbard C. G. Tom L. M. Boussina A. Jin Y. T. Wong H. (2014). Inhibition of microbial sulfate reduction in a flow-through column system by (per)chlorate treatment. *Front. Microbiol.* 5:315. 10.3389/fmicb.2014.00315 25071731 PMC4092371

[B27] Engle M. A. Blondes M. S. (2014). Linking compositional data analysis with thermodynamic geochemical modeling: oilfield brines from the Permian Basin, USA. *J. Geochem. Explor.* 141 61–70. 10.1016/j.gexplo.2014.02.025

[B28] Engle M. A. Reyes F. R. Varonka M. S. Orem W. H. Ma L. Ianno A. J. (2016). Geochemistry of formation waters from the Wolfcamp and “Cline” shales: insights into brine origin, reservoir connectivity, and fluid flow in the Permian Basin, USA. *Chem. Geol.* 425 76–92. 10.1016/j.chemgeo.2016.01.025

[B29] Engle M. A. Rowan E. L. (2013). Interpretation of Na-Cl-Br systematics in sedimentary basin brines: comparison of concentration, element ratio, and isometric log-ratio approaches. *Math. Geosci.* 45 87–101. 10.1007/s11004-012-9436-z

[B30] Gaswirth S. B. Marra K. R. Cook T. A. Charpentier R. R. Gautier D. L. Higley D. K. (2013). *Assessment of Undiscovered Oil Resources in the Bakken and Three Forks Formations, Williston Basin Province, Montana, North Dakota, and South Dakota.* Washington, DC: U.S. Geological Survey, U.S Department of the Interior.

[B31] Gieg L. M. Jack T. R. Foght J. M. (2011). Biological souring and mitigation in oil reservoirs. *Appl. Microbiol. Biotechnol.* 92 263–282. 10.1007/s00253-011-3542-6 21858492

[B32] Gregory K. B. Vidic R. D. Dzombak D. A. (2011). Water management challenges associated with the production of shale gas by hydraulic fracturing. *Elements* 7 181–186. 10.2113/gselements.7.3.181 28159795

[B33] Haluszczak L. O. Rose A. W. Kump L. R. (2013). Geochemical evaluation of flowback brine from Marcellus gas wells in Pennsylvania, USA. *Appl. Geochem.* 28 55–61. 10.1016/j.apgeochem.2012.10.002

[B34] Hitchon B. (1996). Rapid evaluation of the hydrochemistry of a sedimentary basin using only ‘standard’ formation water analyses: example from the Canadian portion of the Williston Basin. *Appl. Geochem.* 11 789–795. 10.1016/S0883-2927(96)00043-1

[B35] Horner R. M. Harto C. B. Jackson R. B. Lowry E. R. Brandt A. R. Yeskoo T. W. (2016). Water use and management in the bakken shale oil play in north Dakota. *Environ. Sci. Technol.* 50 3275–3282. 10.1021/acs.est.5b04079 26866674

[B36] Hull N. M. Rosenblum J. S. Robertson C. E. Harris J. K. Linden K. G. (2018). Succession of toxicity and microbiota in hydraulic fracturing flowback and produced water in the Denver-Julesburg Basin. *Sci. Total Environ.* 644 183–192. 10.1016/j.scitotenv.2018.06.067 29981518

[B37] Iampen H. T. Rostron B. J. (2000). Hydrogeochemistry of pre-mississippian brines, williston Basin, Canada-USA. *J. Geochem. Explor.* 69–70 29–35. 10.1016/S0375-6742(00)00007-8

[B38] Jiménez N. Richnow H. H. Vogt C. Treude T. Krüger M. (2016). Methanogenic hydrocarbon degradation: evidence from field and laboratory studies. *J. Mol. Microbiol. Biotechnol.* 26 227–242. 10.1159/000441679 26959375

[B39] Johnson K. French K. Fichter J. K. Oden R. (2008). *Use of Microbiocides in Barnett Shale Gas well Fracturing Fluids to Control Bacteria Related Problems.* New Orleans, LA: NACE International.

[B40] Korenblum E. Souza D. B. Penna M. Seldin L. (2012). Molecular analysis of the bacterial communities in crude oil samples from two Brazilian offshore petroleum platforms. *Int. J. Microbiol.* 2012 1–8. 10.1155/2012/156537 22319534 PMC3272810

[B41] Kurz B. A. Stepan D. J. Glazewski K. A. Stevens B. G. Doll T. E. Kovacevich J. T. (2016). *A Review of Bakken Water Management Practices and Potential outlook.* Grand Forks, ND: The Energy & Environmental Research Center (EERC).

[B42] Liang R. Davidova I. A. Marks C. R. Stamps B. W. Harriman B. H. Stevenson B. S. (2016). Metabolic capability of a predominant halanaerobium sp. in hydraulically fractured gas wells and its implication in pipeline corrosion. *Front. Microbiol.* 7:988. 10.3389/fmicb.2016.00988 27446028 PMC4916785

[B43] Lipus D. Roy D. Khan E. Ross D. Vikram A. Gulliver D. (2018). Microbial communities in Bakken region produced water. *FEMS Microbiol. Lett.* 365:fny107. 10.1093/femsle/fny107 29688457

[B44] Lipus D. Vikram A. Hammack R. Bibby K. Gulliver D. (2019). The effects of sample storage conditions on the microbial community composition in hydraulic fracturing produced water. *Geomicrobiol. J.* 36 630–638. 10.1080/01490451.2019.1599470

[B45] Lipus D. Vikram A. Ross D. Bain D. Gulliver D. Hammack R. (2017). Predominance and metabolic potential of halanaerobium spp. in produced water from hydraulically fractured marcellus shale wells. *Appl. Environ. Microbiol.* 83:e002659-16. 10.1128/AEM.02659-16 PMC5377500 28159795

[B46] Maeda H. Fujimoto C. Haruki Y. Maeda T. Kokeguchi S. Petelin M. (2003). Quantitative real-time PCR using TaqMan and SYBR Green for *Actinobacillus actinomycetemcomitans*, *Porphyromonas gingivalis*, *Prevotella intermedia*, tetQ gene and total bacteria. *FEMS Immunol. Med. Microbiol.* 39 81–86. 10.1016/S0928-8244(03)00224-4 14557000

[B47] Mohan A. M. Bibby K. J. Lipus D. Hammack R. W. Gregory K. B. (2014). The functional potential of microbial communities in hydraulic fracturing source water and produced water from natural gas extraction characterized by metagenomic sequencing. *PLoS One* 9:e107682. 10.1371/journal.pone.0107682 25338024 PMC4206270

[B48] Murali Mohan A. Hartsock A. Bibby K. J. Hammack R. W. Vidic R. D. Gregory K. B. (2013). Microbial community changes in hydraulic fracturing fluids and produced water from shale gas extraction. *Environ. Sci. Technol.* 47 13141–13150. 10.1021/es402928b 24088205

[B49] Nealson K. H. Scott J. (2006). “Ecophysiology of the Genus Shewanella,” in *The Prokaryotes*, eds Dworkin M. Falkow S. Rosenberg E. Schleifer K.-H. Stackebrandt E. (New York: Springer), 1133–1151. 10.1007/0-387-30746-X_45

[B50] Neuwirth E. (2014). *RColorBrewer: ColorBrewer Palettes. R Package Version 1.1-2.* Available online at: https://CRAN.R-project.org/package = RColorBrewer (accessed January 1, 2020).

[B51] Oksanen J. Blanchet F. G. Kindt R. Legendre P. Minchin R. O’Hara R. B. (2012). *Vegan: Community Ecology Package.*

[B52] Orangi A. Nagarajan N. R. Honarpour M. M. Rosenzweig J. J. (2011). “Unconventional shale oil and gas-condensate reservoir production, impact of rock, fluid, and hydraulic fractures,” in *Proceedings of the SPE Hydraulic Fracturing Technology Conference*, Woodlands, TX.

[B53] Osselin F. Saad S. Nightingale M. Hearn G. Desaulty A.-M. Gaucher E. C. (2019). Geochemical and sulfate isotopic evolution of flowback and produced waters reveals water-rock interactions following hydraulic fracturing of a tight hydrocarbon reservoir. *Sci. Total Environ.* 687 1389–1400. 10.1016/j.scitotenv.2019.07.066 31412472

[B54] Paukert Vankeuren A. N. Hakala J. A. Jarvis K. Moore J. E. (2017). Mineral reactions in shale gas reservoirs: barite scale formation from reusing produced water as hydraulic fracturing fluid. *Environ. Sci. Technol.* 51 9391–9402. 10.1021/acs.est.7b01979 28723084

[B55] Pedregosa F. Varoquaux G. Gramfort A. Michel V. Thirion B. Grisel O. (2011). Scikit-learn: machine learning in python. *Mach. Learn. Python* 6 2826–2830.

[B56] Pitman J. K. Price L. C. LeFever J. A. (2001). *Diagenesis and Fracture Development in the Bakken Formation, Williston Basin: Implications for Reservoir Quality in the Middle Member.* Reston: U.S. Department of the Interior, U.S. Geological Survey.

[B57] Plummer M. Wood T. Huang H. Guo L. Reiten J. Chandler K. (2013). Water needs and availability for hydraulic fracturing in the bakken formation, Eastern Montana. *EPA* 2:21.

[B58] Quast C. Pruesse E. Yilmaz P. Gerken J. Schweer T. Yarza P. (2012). The SILVA ribosomal RNA gene database project: improved data processing and web-based tools. *Nucleic Acids Res.* 41 D590–D596. 10.1093/nar/gks1219 23193283 PMC3531112

[B59] R Core Team (2013). *R: A Language And Environment For Statistical Computing.* Vienna: R Foundation for Statistical Computing.

[B60] Rivard C. Lavoie D. Lefebvre R. Séjourné S. Lamontagne C. Duchesne M. (2014). An overview of Canadian shale gas production and environmental concerns. *Intern. J. Coal Geol.* 126 64–76. 10.1016/j.coal.2013.12.004

[B61] Stepan D. J. Shockey R. E. Kurz B. A. Kalenze N. S. Cowan R. M. Ziman J. J. (2010). *Bakken Water Opportunities Assessment – Phase 1.* Grand Forks, ND: University of North Dakota, Energy & Environmental Research Center.

[B62] Stevens P. (2013). Shale gas in the United Kingdom. *Energy Environ. Resour.* 10 1–10.

[B63] Stringfellow W. T. Domen J. K. Camarillo M. K. Sandelin W. L. Borglin S. (2014). Physical, chemical, and biological characteristics of compounds used in hydraulic fracturing. *J. Hazard. Mater.* 275 37–54. 10.1016/j.jhazmat.2014.04.040 24853136

[B64] Strong L. C. Gould T. Kasinkas L. Sadowsky M. J. Aksan A. Wackett L. P. (2014). Biodegradation in waters from hydraulic fracturing: chemistry, microbiology, and engineering. *J. Environ. Eng.* 140:792. 10.1061/(ASCE)EE.1943-7870.0000792 29515898

[B65] Struchtemeyer C. G. Elshahed M. S. (2012). Bacterial communities associated with hydraulic fracturing fluids in thermogenic natural gas wells in North Central Texas, USA. *FEMS Microbiol. Ecol.* 81 13–25. 10.1111/j.1574-6941.2011.01196.x 22066833

[B66] Struchtemeyer C. G. Morrison M. D. Elshahed M. S. (2012). A critical assessment of the efficacy of biocides used during the hydraulic fracturing process in shale natural gas wells. *Intern. Biodeterior. Biodegrad.* 71 15–21. 10.1016/j.ibiod.2012.01.013

[B67] Tanveer S. Chen C. (2020). A comprehensive thermodynamic model for high salinity produced waters. *AIChE J.* 66:18 10.1002/aic.16818

[B68] Tatar A. (2018). “Microbial enhanced oil recovery,” in *Fundamentals of Enhanced Oil and Gas Recovery from Conventional and Unconventional Reservoirs* (Amsterdam: Elsevier), 291–508. 10.1016/B978-0-12-813027-8.00010-2

[B69] Thyne G. Brady P. (2016). Evaluation of formation water chemistry and scale prediction: bakken shale. *Appl. Geochem.* 75 107–113. 10.1016/j.apgeochem.2016.10.015

[B70] Torres L. Yadav O. P. Khan E. (2016). A review on risk assessment techniques for hydraulic fracturing water and produced water management implemented in onshore unconventional oil and gas production. *Sci. Total Environ.* 539 478–493. 10.1016/j.scitotenv.2015.09.030 26386446

[B71] Toth C. R. A. Gieg L. M. (2018). Time course-dependent methanogenic crude oil biodegradation: dynamics of fumarate addition metabolites, biodegradative genes, and microbial community composition. *Front. Microbiol.* 8:2610. 10.3389/fmicb.2017.02610 29354103 PMC5758579

[B72] U.S. Department of Energy, Energy Information Administration, and Independent Statistics & Analysis (2020a). *Hydraulically Fractured Horizontal Wells Account For Most New Oil And Natural Gas Wells.* Available online at: https://www.eia.gov/todayinenergy/detail.php?id=34732 (accessed March 6, 2020).

[B73] U.S. Department of Energy, Energy Information Administration, and Independent Statistics & Analysis Annual Energy Review (2020b). Available online at: https://www.eia.gov/totalenergy/data/annual/ (accessed January 8, 2020).

[B74] U.S. Department of Energy, Energy Information Administration, and Independent Statistics & Analysis Drilling Productivity Report (2020c). Available online at: https://www.eia.gov/petroleum/drilling/ (accessed January 9, 2020).

[B75] Vengosh A. Kondash A. Harkness J. Lauer N. Warner N. Darrah T. H. (2017). The geochemistry of hydraulic fracturing fluids. *Proc. Earth Planet. Sci.* 17 21–24. 10.1016/j.proeps.2016.12.011

[B76] Vigneron A. Alsop E. B. Lomans B. P. Kyrpides N. C. Head I. M. Tsesmetzis N. (2017). Succession in the petroleum reservoir microbiome through an oil field production lifecycle. *ISME J.* 11 2141–2154. 10.1038/ismej.2017.78 28524866 PMC5563965

[B77] Vikram A. Bomberger J. M. Bibby K. J. (2015). Efflux as a glutaraldehyde resistance mechanism in *Pseudomonas fluorescens* and *Pseudomonas aeruginosa* biofilms. *Antimicrob. Agents Chemother.* 59 3433–3440. 10.1128/AAC.05152-14 25824217 PMC4432172

[B78] Vikram A. Lipus D. Bibby K. (2014). produced water exposure alters bacterial response to biocides. *Environ. Sci. Technol.* 48 13001–13009. 10.1021/es5036915 25279933

[B79] Wang H. Lu L. Chen X. Bian Y. Ren Z. J. (2019). Geochemical and microbial characterizations of flowback and produced water in three shale oil and gas plays in the central and western United States. *Water Res.* 164:114942. 10.1016/j.watres.2019.114942 31401327

[B80] Yilmaz P. Parfrey L. W. Yarza P. Gerken J. Pruesse E. Quast C. (2014). The SILVA and “All-species living tree project (LTP)” taxonomic frameworks. *Nuclear Acids Res.* 42 D643–D648. 10.1093/nar/gkt1209 24293649 PMC3965112

