# Supplementary material for: Geochemistry and Microbiology Predict Environmental Niches With Conditions Favoring Potential Microbial Activity in the Bakken Shale
Source: Front Microbiol. 2020 Jul 30;11:1781. doi: 10.3389/fmicb.2020.01781 (PMC7406717; doi:10.3389/fmicb.2020.01781)
Supplement: Supplementary file 1 [file Data_Sheet_1.PDF]

Supplementary Information

SI Data 1. gBlocks Gene Fragment Sequence for qPCR Standard. Fragment was constructed using the 16S bac, McrA, DsrA, and RdlA genes.

ACAAAGAAGCCCACAGCCGACTTGGTTTCTACGGTTACGACCTGCAGGACCAGTGCGGAGCATCCAACTCTCTCTC
CATCAGGAGTGACGAAGGTCTGATCCACGAATTACGTGGTCCTAACTACCCTAACTACGCCATGAAAAACGCTGGT
CTCTCCGCATGGTATCTCTGTATGTACCTGCACAAGGAAGGTCACGGACGTCTGGGATTCTTCGGATTCGACTTGC
AGGACCAGTGTGGTGCAACCAACACCTTCTCCTACCAATCCGACGAAGGTCTTGATGACTTGACGTCATCCCCACC
TTCCTCCCCGTTAACCGGGGCGGTCTCCATAGAGTGCCCAGCATTACCTGGTAGCAACTATGAACAGGGGTTGCGC
TCGTTGCGGGACTTAACCCAACACCTCACGGCACGAGCTGACGACAGCCATGCAGCACCTGTCTCGGGGGCGTCA
TCGGCCGTTACTGTGACCAGCCCGAACAGTTCCCCGGCGTGGCGCACTTCCACACCGTGCGCGTGAACCAGCCCGC
GGCGAAGTACTACCACACCGACTACCTGCGCCAGCTCTGCGACCTGTGGGACCTGCGCGGCTCCGGTCTGACCAA
CATGCACGGTTCCACCGGCGACATCGAGTATTTTGGACAGCTGCCTCAGGATAAGAAGATAGTTGTTTACTGCTAT
ACAGGTCAAACAGCAGGTCAGGCAGTAGCAGGTTTAAGAATGTTAGACTATGATGCTGTATCACTTAATGGTGGT
ATGGGTACTTCTGCTAATGAGCCT

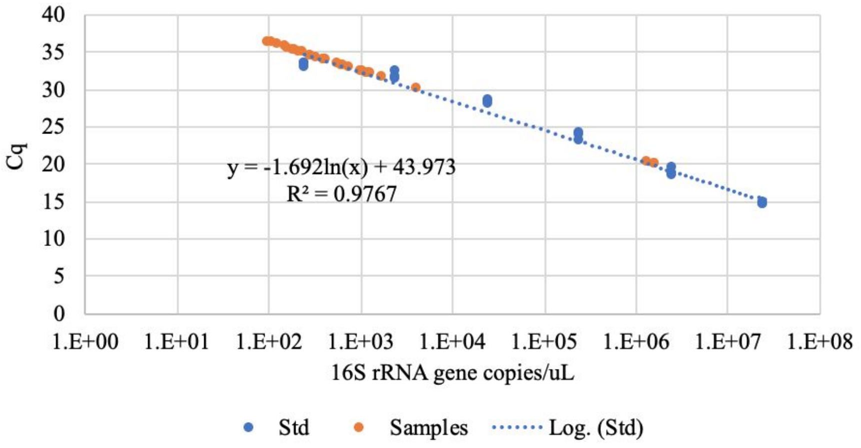

SI Figure 1. qPCR standard curve. Sample extraction volumes were normalized in order to calculate 16S rRNA gene copies/mL.

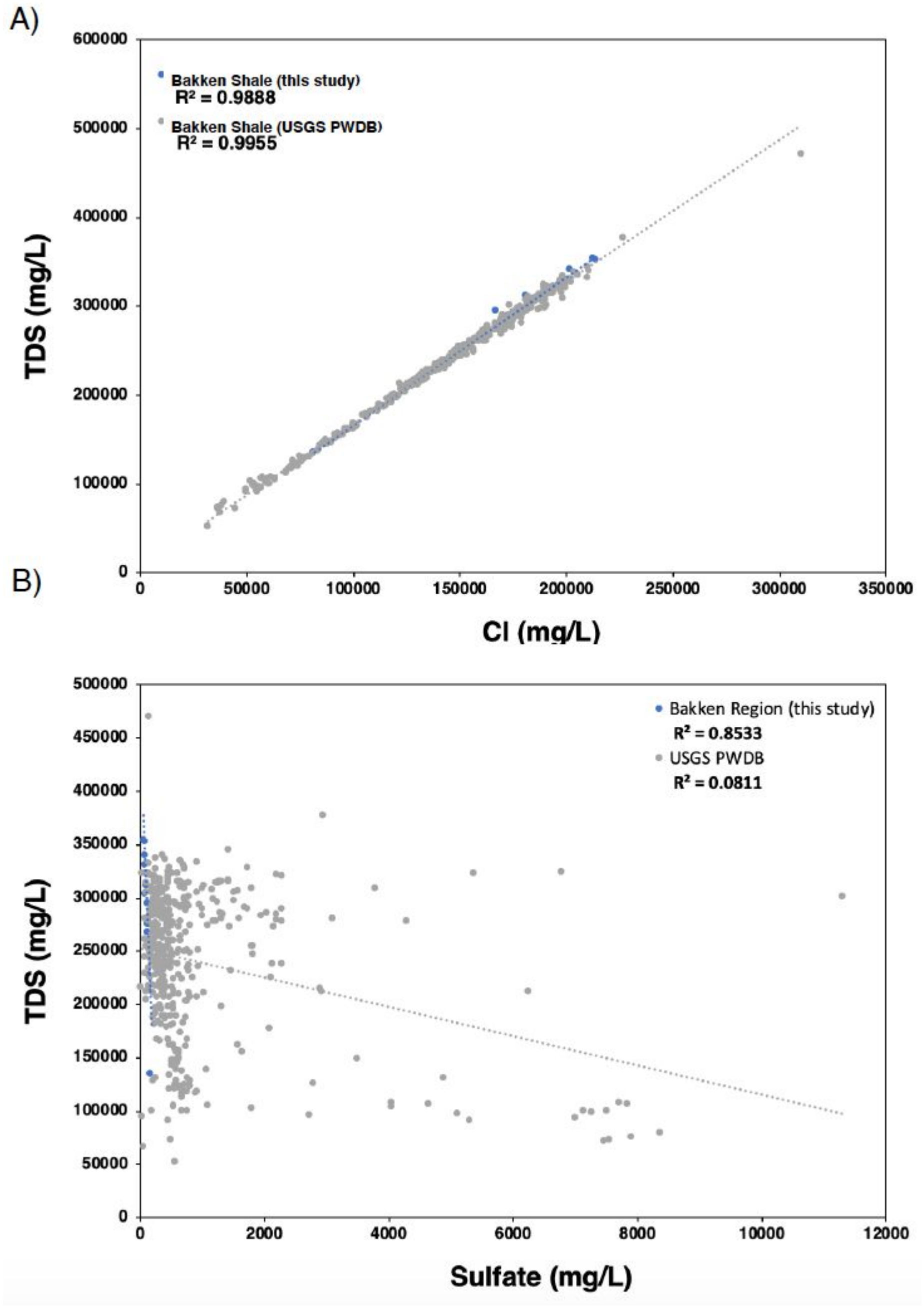

SI Figure 2. A) TDS and chloride measurements plotted for this study and for similar produced waters found in the USGS PWDB (Blondes et al., 2019). Linear trendlines report high linear correlation values, displaying that chloride is the primary anion and well 11, with its lower TDS value, does not have an anomalous chloride value. The trendline excludes well 18, a sample that, based on its geochemistry and age, likely represents a mix of flowback and produced water. B) TDS and sulfate measurements plotted for produced waters in this study and for similar produced waters found in the USGS PWDB (Blondes et al., 2019). For samples in this study, there is a higher linear association (R^2=0.8533) for these analytes when the youngest and spatially unique sample, well 18, is excluded. Based on measurements from this study and previous research, the elevated sulfate in well 18 could be related to residual chemicals from hydraulic fracturing operations or the product of flowback waters.

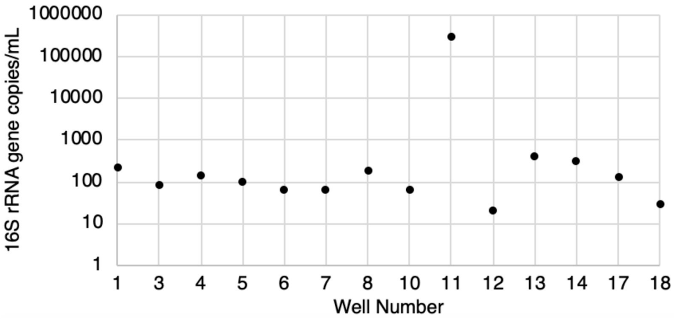

SI Figure 3. Microbial abundances for each produced water sample.

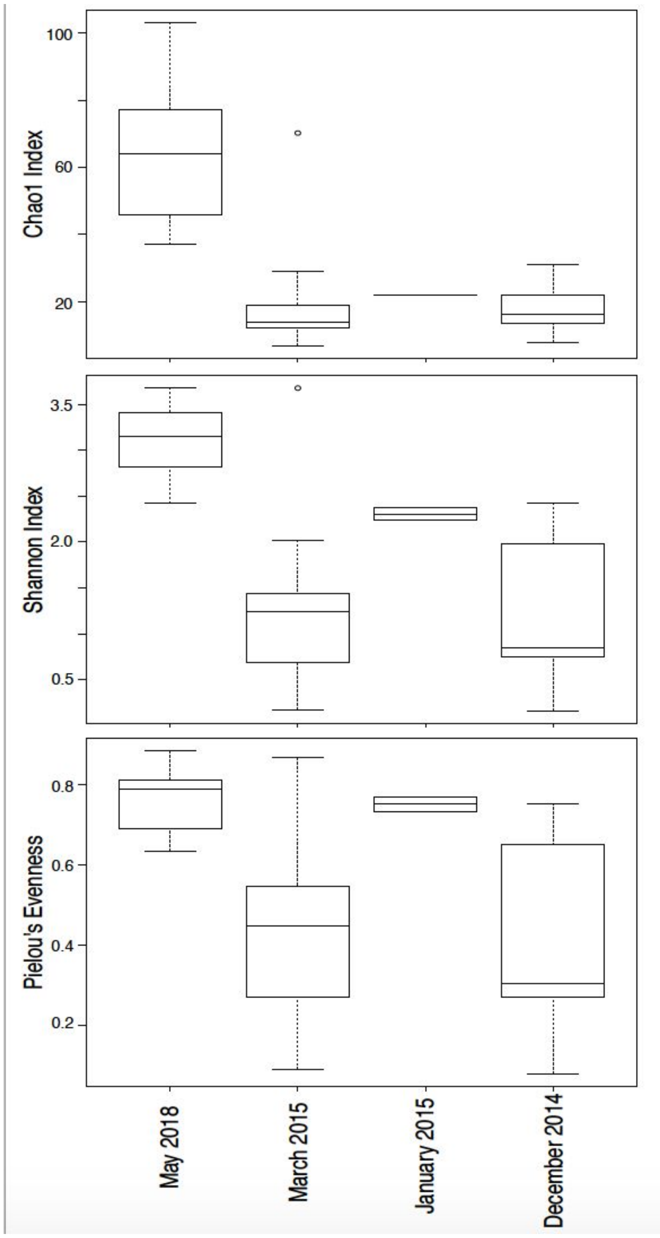

SI Figure 4. Boxplots of alpha diversity measurements within produced water samples. Boxplots show Chao1 Richness (top), Shannon Diversity (middle), and Pielou's Eveness (bottom) in our samples (May 2018) and those collected by Lipus et al. in March 2015, January 2015, and December 2014. All alpha diversity measurements are shown in SI Table 1 and were calculated after resampling libraries to a depth of 1942. For each group, the bars delineate the median, the hinges represent the lower and upper quartiles, the whiskers extend to the lesser of the most extreme value or 1.5 times the interquartile range, and outliers are plotted, if present.

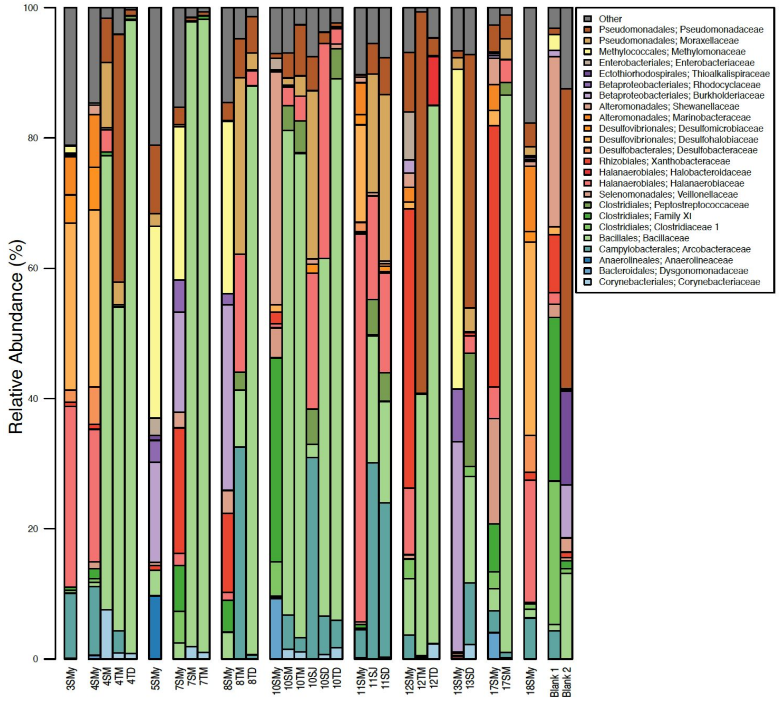

SI Figure 5. Relative abundance of microbial families in produced water samples. Samples from our current study are shown next to samples previously collected from the same site (Lipus et al., 2018). Two of the 10 blanks that resulted in sequencing data are also shown. Samples are clustered by well number, sampling location (S: Separator, T: Tank), and sample event (My: May 2018; M: March 2015; J: January 2015; D: December 2014; O: October 2014). All families that represent ≥5% of sequences from any one sample are listed in the barplot, all other families are grouped together under "Other."

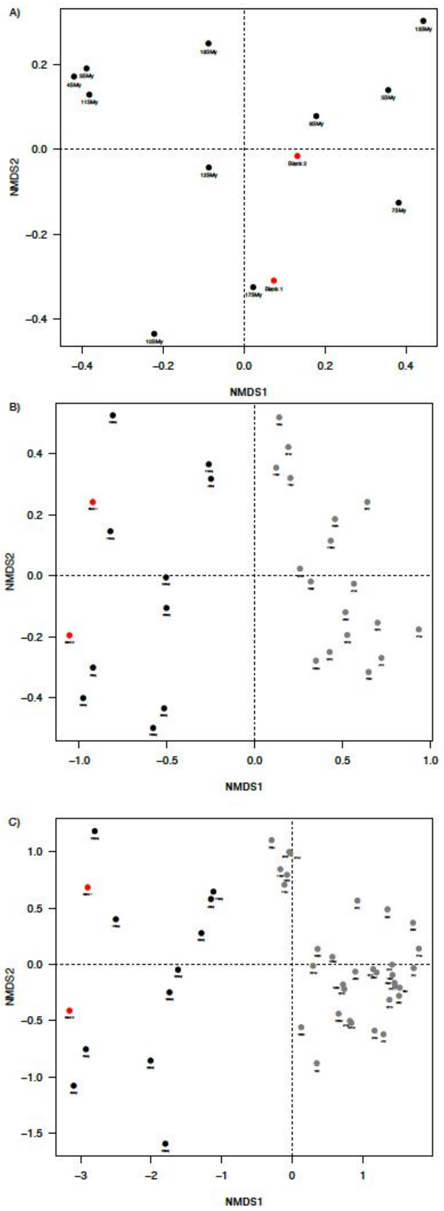

SI Figure 6. Nonmetric multidimensional scaling (NMDS) plots of: A) samples and blanks from our current study, B) samples and blanks from our current study as well as samples previously collected from the same site (Lipus et al., 2018), and C) samples and blanks from our current study and all samples previously collected by Lipus et al. All NMDS plots were constructed using Bray-Curtis distance calculated after sequence libraries were resampled to the depth of the sample with the fewest sequences from this experiment (1942 sequences). Constructed plots had stress values of 0.08576772, 0.07193307, and 0.07895004, respectively. Data from our current study are colored black (experimental data) or red (blanks) while data from Lipus et al. is gray. Each sample is labeled by its corresponding well number, sampling location (S: Separator, T: Tank), and sample event (My: May 2018; M: March 2015; J: January 2015; D: December 2014; O: October 2014).

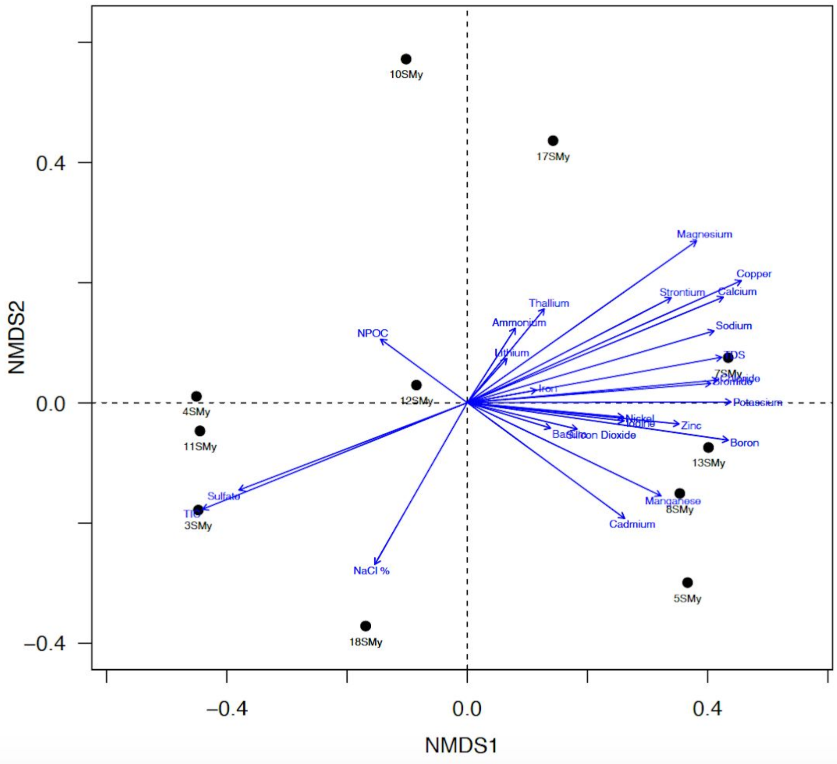

	R^2	p-value
**Boron**	0.6486	0.008*
**Barium**	0.0707	0.757
**Bromide**	0.557	0.032*
**Calcium**	0.7157	0.005*
**Cadmium**	0.3559	0.166
**Chloride**	0.5917	0.022*
**Copper**	0.8396	0.001*
**Iron**	0.0462	0.849
**Iodine**	0.2323	0.326
**Lithium**	0.0328	0.867
**Magnesium**	0.7345	0.005*
**Manganese**	0.4314	0.113
**Sodium**	0.616	0.006*
**Nickel**	0.2294	0.368
**Ammonium**	0.0734	0.742
**Potassium**	0.6493	0.012*
**Silicon Dioxide**	0.119	0.574
**Strontium**	0.4906	0.059
**Sulfate**	0.558	0.041*
**Thallium**	0.1376	0.564
**Zinc**	0.4229	0.132
**TIC**	0.76	0.001*
**NPOC**	0.1076	0.647
**TDS**	0.6236	0.015*
**NaCl%**	0.3236	0.217

*p-value ≤ 0.05

SI Figure 7. (Left) Nonmetric multidimensional scaling (NMDS) plot of produced water samples with a stress value of 0.07194914. For this plot, sequence libraries were resampled to the depth of the sample with the fewest sequences from this experiment (1942 sequences). Vectors for geochemical parameters fit onto the plot are shown in blue. The direction of the arrow corresponds to the direction of the gradient while the length of the vector is proportional to the correlation between ordination and the environmental variable. (Right) Table containing the R^2 and p-values for all constructed environmental vectors.
